# miR-1227-3p participates in the development of fetal growth restriction via regulating trophoblast cell proliferation and apoptosis

**DOI:** 10.1038/s41598-022-10127-w

**Published:** 2022-04-16

**Authors:** Jiawen Cui, Xinyi Kang, Yanxing Shan, Mingjin Zhang, Ying Gao, Wei Wu, Liping Chen

**Affiliations:** 1 Obstetrics and Gynecology Department, The Second Affiliated Hospital of Nantong University, No.6, North Road, Haierxiang, Chongchuan District, Nantong, 226001 Jiangsu China; 2 State Key Laboratory of Reproductive Medicine, Institute of Toxicology, Nanjing Medical University, Nanjing, 211166 Jiangsu China; 3 Key Laboratory of Modern Toxicology of Ministry of Education, School of Public Health, Nanjing Medical University, Nanjing, 211166 Jiangsu China; 4 Obstetrics and Gynecology Department, Qingpu Branch of Zhongshan Hospital Affiliated to Fudan University, Qingpu, Shanghai, 201700 China

**Keywords:** Developmental biology, Predictive markers

## Abstract

Fetal growth restriction (FGR) is a common obstetric disease, which is harmful to the pregnant women and fetuses. It has many influencing factors, but the specific etiology is not clear. MiRNA plays an important role in the fetal growth and development. In this article, we use TaqMan Low-Density Array to screen and analyze the differently expressed miRNAs in FGR-affected placenta (n = 40) and the normal placenta (n = 40). A total of 139 abnormally expressed miRNAs in the FGR-affected placenta were identified, and miR-1227-3p was the most highly downregulated miRNA. Importantly, miR-1227-3p may promote the proliferation in HTR-8/SVneo cells, while inhibited the apoptosis of HTR-8/SVneo cells. DAVID was used to analyze the pathway enrichment of target genes of miR-1227-3p to predict its mechanism of action. Furthermore, the putative targets of miR-1227-3p were predicted using the TargetScan, PicTar, DIANA LAB, and miRWalk database. The potential expression of target genes of miR-1227-3p, including *PRKAB2*, *AKT1*, *PIK3R3*, and *MKNK1* were significantly increased in FGR-affected placenta. Taken together, miR-1227-3p may participate in the development of FGR via regulating trophoblast cell proliferation and apoptosis by targeting genes involved in the insulin pathway. MiR-1227-3p may have a potential clinical value in the prevention and treatment of FGR, we need to study further to prove its value in the future.

## Introduction

Fetal growth restriction (FGR) is a common disorder in obstetrics and causes many adverse effects on pregnant women and fetuses, such as premature birth, fetal distress, and serious fetal death^1^. In relatively developed countries, FGR affects 5–10% of pregnancies and should be responsible for 30% of fetal deaths^2^. Maternal nutrition supply, material transport capacity of placenta, and genetic factors are the three major causes of FGR^3^. Accumulating evidence suggests that about 40% of FGR are idiopathic cases with unknown causes, and the pathological basis of this part of FGR is mostly placental trophoblast dysfunction^4^. However, the molecular mechanisms of trophoblast dysfunction-induced FGR remain unclear^5,6^. Importantly, the inadequacy of FGR therapy emphasizes the need for a better understanding of how fetal development is dysregulated in FGR.

MicroRNA (miRNA) belongs to non-coding RNA on average 22 nucleotides in length and has been shown to be associated with multiply diseases development^7–10^. Growing evidence indicates the important role of miRNA in pathological pregnancy and fetal growth and development, thus miRNA becomes a hot topic in the research of perinatal medicine^11^. Evidence shows that the abnormal expression of multiple miRNAs in placental tissue is significantly related to fetal growth and development^12^. Tang et al. demonstrated that aberrant high expression level of miR-141 might play important roles in the pathogenesis of FGR by suppressing E2F3 and PLAG1^13^. Furthermore, The expression of six miRNAs in C19MC gene cluster was down regulated (miR-517-5p, miR-518f-5p, miR-519a, miR-519d, miR-520a-5p and miR-525). The expression of miR-519d decreased significantly. In another study, the expression of miR-518b decreased in placenta of pregnant women with FGR, while the expression of miR-519a increased significantly^14,15^. However, the expression profile of miRNAs and the role of the most aberrantly expressed miRNA in FGR are still unknown.

The priority of this work was to identify the differentially expressed miRNAs in FGR through microarray, investigate the target genes, and illustrate the function of miR-1227-3p on HTR-8/SVneo cells. Our findings will lay a foundation for future etiological research and clinical technology development of FGR, and provide a potential biomarker for the disease treatment.

## Methods

### Tissues specimens

The study was authorized by the Second Affiliated Hospital of Nantong University Ethics Committee (No. 2017-008). We have obtained informed consent signed by all pregnant women before participation. All clinical investigations were required to complying with the principles of the Declaration of Helsinki. A total of 80 pregnant women (FGR and normal groups, n = 40 per group), who delivered at the Second Affiliated Hospital of Nantong University between May 2017 to April 2018, participated in this research. Inclusion criteria: pregnancy (> 37 weeks), 18–40 years old, number of pregnancies (< 3 times), BMI 18–25 kg/m^2^ before pregnancy, single pregnancy and first labor. Exclusion criteria: patients with gestational diabetes mellitus, gestational hypertension, chronic kidney disease, thyroid disease, placenta previa, placental abruption, smoking, drinking, and other serious diseases. FGR diagnostic criteria refer to the 8th edition of Obstetrics and Gynecology^16^.

Placental tissues with a size of 1 × 1 × 1 cm were quickly and randomly obtained from the region near the root of the umbilical cord and maternal surface of the placenta after delivery. Three pieces of tissues were detached from the same placenta. Subsequently, all placenta tissues were cleared using the ice-code sterile saline three times to remove blood, and then frozen in liquid nitrogen immediately followed by storing at − 80 °C.

### Cell culture

HTR-8/SVneo, the human extravillous trophoblast cell line, was obtained from the Department of Gynecology and Obstetrics (Affiliated Drum Tower Hospital of Nanjing University Medical School, Nanjing, China). RPMI-1640 medium was purchased from Gibco Life Technologies (Carlsbad, CA), and then supplemented with 10% fetal bovine serum (Gibco Life Technologies), 100 U/mL penicillin, and 100 U/mL streptomycin for culturing HTR-8/SVneo cells at 37 °C in an incubator with 5% CO_2_.

### miRNA TLDA analysis and qRT-PCR assay

Total RNA was isolated from the human placenta tissues and the cultured cells using the TRIzol reagent (Carlsbad, CA, USA). NanoDrop^®^ ND-1000 was used to examine the concentration and purity of total RNA. To detect the generalizable signatures of miRNAs in FGR, we pooled placenta RNA samples of 10 FGR and 10 normal controls, respectively and subjected them to TaqMan Low Density Array (TLDA, TaqMan^®^ Array Human MicroRNA A + B Cards Set v3.0, Applied BiosystemsInc., CA, USA) screening. Megaplex RT reactions and pre-amplification reactions were run according to the manufacturer’s protocol. Then, we investigated the expression profiling of miRNAs in the placenta tissues utilizing the TLDA chip on a 7900HT instrument (Applied Biosystems). U6 was served as the internal reference of the target miRNAs (△Ct = Ct_sample_ − Ct_U6_).

For the qRT-PCR assay, total RNA was reverse transcribed into cDNA using the PrimeScript RT reagent Kit (Takara), and then qPCR analysis was carried out using the SYBR^®^ Premix Ex Taq™ (Takara) on an ABI Prism 7900HT (Applied Biosystems). All experiments were implemented under the corresponding instructions. U6 and GAPDH were used as the standard to normalize the expression level of miRNA and mRNA, respectively. The relative gene expression levels were calculated using the 2^−△Ct^ method. All the reactions were run in triplicate to control for PCR variation. The primer sequences for qRT-PCR were listed in Table 1.

**Table 1:** The primer sequences in qRT-PCR assay.

Gene	Primers	Sequence(5′− → − 3′)
PRKAB2	Forward	TCCCTTGCATTTCTGGACTGA
	Reverse	CAGAGCATCCTACTCAGAGGC
AKT1	Forward	CAGCCCTCAGAACAATCCGA
	Reverse	ATAGCTGGTGACAGACAGCC
PIK3R3	Forward	TGAACGATGGCTCAATCACA
	Reverse	TAATGGGGCAGGTTTTCATCT
MKNK1	Forward	GATGTCCTCTTTGCCCGTCT
	Reverse	TGCAGACCGGAAGACTGATT
SOS1	Forward	CACCTCCTCCTCAAACACCT
	Reverse	GTGTGTGTGCTCCCTTTTGT
PKM2	Forward	TGTGGCTCGTCTGAACTTCT
	Reverse	GGCGTTATCCAGCGTGATTT
PRKX	Forward	GATGCTTTCGGGGTTTCCTC
	Reverse	GGAACCACCGATGATGCTTC
PRKAA2	Forward	GGCCTGAAACCTCATCCAGA
	Reverse	AGCTCGGTAAACTTCAGCCA
SOCS3	Forward	CACCTACTGAACCCTCCTCC
	Reverse	AGAGATGCTGAAGAGTGGCC
miR-1227-3p	Forward	GAACGCGCTTCCCTAT
	Reverse	CAGTGCGTGTCGTGGAG
GADPH	Forward	GCACCGTCAAGGCTGAGAAC
	Reverse	GGATCTCGCTCCTGGAAGATG
U6	Forward	CTCGCTTCGGCAGCACA
	Reverse	AACGCTTCACGAATTTGCGT

### Cell transfection

Mimics negative control (mimics NC), miR-1227-3p mimics, inhibitor negative control (NC-In), or miR-1227-3p inhibitor (miR-1227-3p-In) were purchased from Genepharma (GenePharma, Shanghai, China). Transient transfection was performed using Lipofectamine 2000 reagents (Invitrogen, CA, USA) according to the manufacture’s protocol.

### Cell proliferation assay

The proliferation of HTR-8/SVneo cells was assayed using Cell Counting Kit-8 (CCK-8, Dojindo, Kumamoto, Japan). All operations were carried out following with the instructions of the manufacturer. In brief, HTR-8/SVneo cells with a density of 1 × 10^3^ cells/well were seeded into 96-well plates. After 24 h of transfection, 10 μl per well of CCK-8 solution was added into 96-well plates. The plates were maintained at 37 °C in an incubator for 2 h. Subsequently, the absorbance at 450 nm was measured using the TECAN infinite M200 Multimode microplate reader (Tecan, Mechelen, Belgium). The experiments were repeated thrice independently.

### Cell apoptosis assay

The apoptotic rate of HTR-8/SVneo cells was monitored using an Annexin V-FITC Kit (BD Pharmingen, NJ, USA) by flow cytometry (Becton Dickinson Immunocytometry Systems, San Jose, CA, USA). Cells with a density of 4 × 10^6^ cells/well were plated into 6-well plates, and then transfected with NC, miR-1227-3p, NC-In and miR-1227-3p-In, respectively. After 24 h, ice-code 0.01 M PBS was used to wash the cells twice. Subsequently, cells were maintained with Annexin V-FITC for 15 min at room temperature and in the darkness. Then, the apoptotic cells were inspected using FACS Calibur Flow Cytometer (BD Biosciences, USA) immediately.

### Bioinformatics analysis

Bioinformatic analysis was performed using the OmicStudio tools at https://www.omicstudio.cn/tool. The possible target genes of miR-1227-3p were predicted using four databases, including DIANA LAB (http://diana.imis.athena-innovation.gr/DianaTools/index.php), PicTar (http://pictar.mdc-berlin.de/), TargetScan (http://www.targetscan.org/vert_71/), and miRWalk database (http://mirwalk.umm.uni-heidelberg.de/). The Kyoto Encyclopedia of Genes and Genomes (KEGG) (http://www.genome.jp/kegg/) pathway enrichment analysis was implemented using DAVID tools (https://david.ncifcrf.gov/home.jsp).

### Statistical analysis

Statistical analyses were carried out using the statistical software of SPSS 24.0 (IBM Corp., Armonk, NY, USA) and GraphPad Prism 5.01 (GraphPad Software Inc., San Diego, CA). The categorical variables were expressed by frequency and rate, the classification variables were expressed by the number of cases (percentage), and the comparison between groups was expressed by χ^2^ test. If data were in normal distribution, the student's t test was used to compare two groups. Otherwise, the Mann–Whitney U-test was used. *P* < 0.05 was considered statistically significant.

## Results

### Clinical characteristics of pregnant women

The clinical characteristics of the two groups were summarized in Table 2. In these data, compared with the control group, the weight gain during pregnancy and birth weight of newborns were significantly lower in the FGR group (*P* < 0.05). However, no significant difference was found in maternal age, gestational age, body mass index of pre-pregnancy, or the gender of newborns between the FGR group and the control group (*P* > 0.05).

**Table 2:** Clinical characteristics of the study population.

Characteristic	Control (n = 40)	FGR (n = 40)	*P*-values
Maternal age (years)	26.86 ± 2.73	28.37 ± 3.33	0.563
Gestational age (weeks)	37.89 ± 0.97	38.40 ± 0.91	0.168
BMI before pregnancy (kg)	20.15 ± 2.33	20.28 ± 2.62	0.748
Weight gain during pregnancy (kg)	17.69 ± 3.86	15.78 ± 3.78	0.032
Birth weight (g)	3816.59 ± 243.01	2163.67 ± 278.89	< 0.001
**Infant gender, n (%)**			
Male	20 (50.0)	20 (50.0)	
Female	20 (50.0)	20 (50.0)	

Values are mean ± SD.

### Expression levels of miRNAs in human FGR-affected placentas

**Figure 1:**
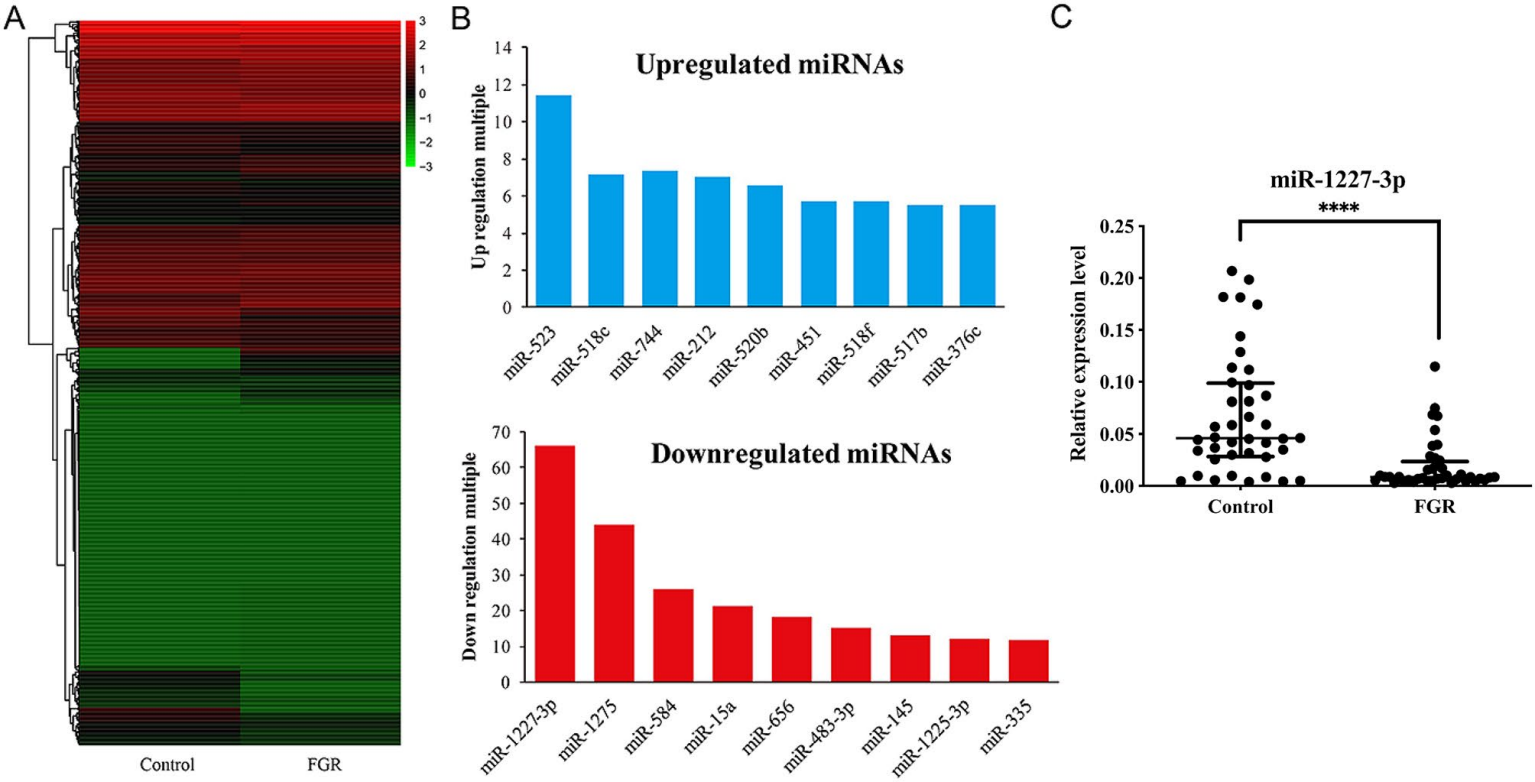
The expression pattern of miRNAs in placenta of FGR. (**A**) Heat map of differentially expressed miRNA in placental tissue of FGR-affected and normal. (**B**) Top nine upregulated miRNAs in FGR-affected placenta, and top nine downregulated miRNAs in FGR-affected placenta. (**C**) The expression of miR-1227-3p in 40 FGR-affected placenta and in 40 normal tissues. Data are given in Tukey Box plots showing median (−) and mean (+) values. Asterisks denote significant differences from controls (****P < 0.0001).

To scrutinize the differential expression profiles of miRNAs between FGR-affected and normal placenta tissues, we randomly selected 10 FGR-affected and 10 normal placenta tissues for miRNA TLDA screening. All the analyzed miRNAs were shown in Supplementary Table S1. In the screening phase, 139 aberrantly expressed miRNAs (40 upregulated and 99 downregulated miRNAs) were identified in FGR-affected placenta tissues with a fold change ≥ 2 (Fig. 1A). Based on both scientific and applicable considerations, we selected miRNAs that had a Ct value less than 30 in both the groups for further analyze. The top 9 upregulated and downregulated miRNAs in FGR-affected placenta tissues were shown in Fig. 1B, respectively. Meanwhile, miR-1227-3p was the most highly downregulated miRNA with an approximately eight-fold change.

### miR-1227-3p were downregulated in FGR-affected placenta tissue

According to the results of miRNA TLDA analysis, we proposed that the abnormally expressed miR-1227-3p might participate in the development of FGR. Hence, we further verified the expression level of miR-1227-3p by qRT-PCR using 40 FGR patients and 40 healthy controls (including the same 10 FGR patients and 10 healthy controls in the screening phase). Our data showed that miR-1227-3p expression was notably downregulated in FGR-affected placenta (Fig. 1C). The expression level of miR-1227-3p in FGR placenta tissue was significantly lower than that in the normal control group (*P* < 0.0001), which is consistent with the result of miRNA TLDA analysis.

### miR-1227-3p promoted trophoblast cell proliferation, while inhibited its apoptosis

**Figure 2:**
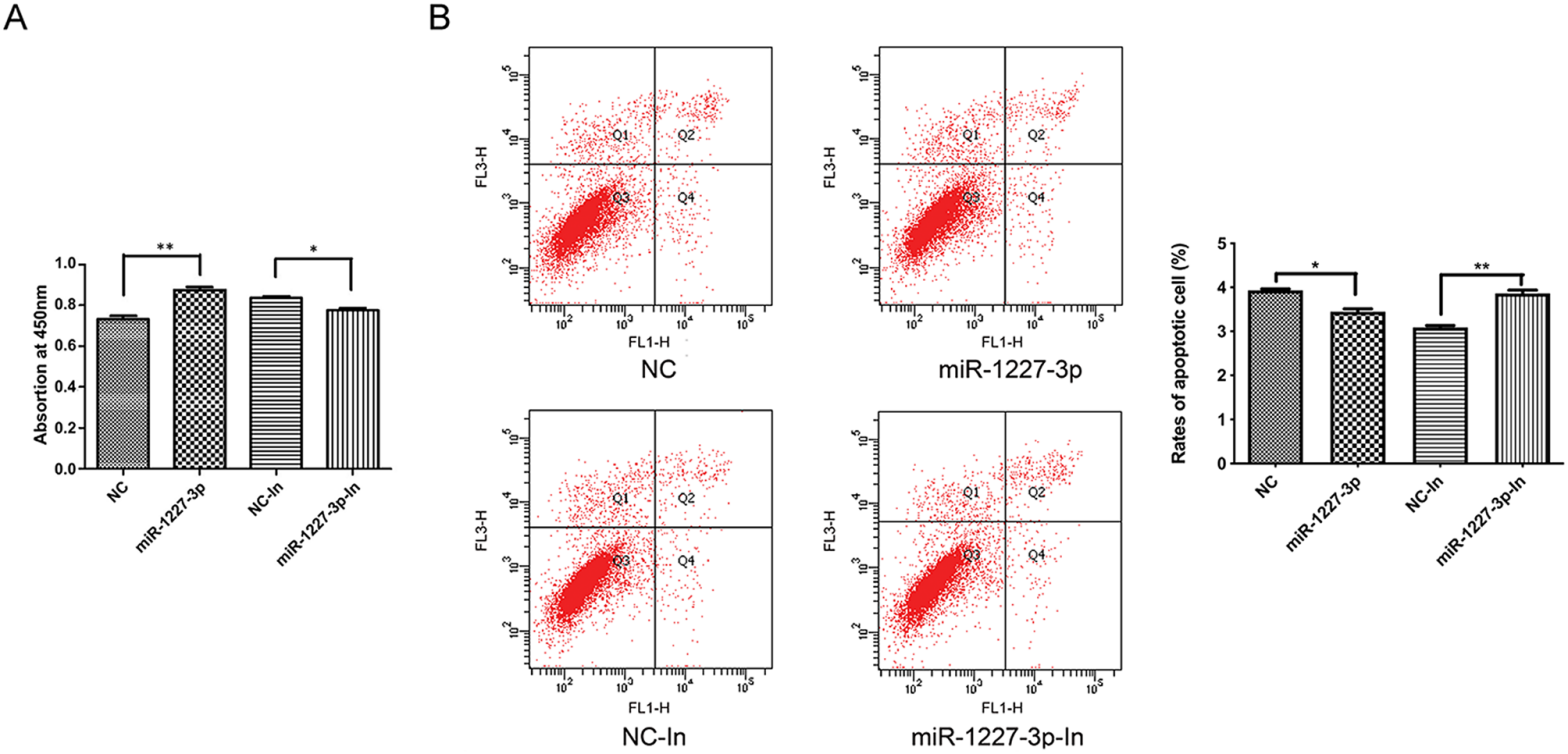
Effect of miR-1227-3p on HTR-8/SVneo cells proliferation and apoptosis. (**A**) CCK-8 assay was performed to detect the proliferation of HTR-8/SVneo cells treated with miR-1227-3p mimics or inhibitor. (**B**) Flow cytometry assay was carried out to examine the cell apoptosis of HTR-8/SVneo cells transfected with miR-1227-3p mimics or inhibitor. Asterisks denote significant differences from controls (*P < 0.05 and **P < 0.01).

Recently, Cao et al. demonstrated that miR-1227-3p served as a tumor suppressor in hepatocellular carcinoma via suppressing the proliferation of tumor cells^17^. Here, we further investigated the role of miR-1227-3p in FGR development. Cell proliferation was monitored by CCK-8 assay, and the results indicated that the overexpression of miR-1227-3p by transfection with miR-1227-3p mimics promoted the proliferation of HTR-8/SVneo cells, and inhibition of miR-1227-3p by transfection with miR-1227-3p inhibitor repressed the proliferation (Fig. 2A). Furthermore, the results of flow cytometry revealed that the upregulation of miR-1227-3p suppressed the apoptosis of HTR-8/SVneo cells, and the downregulation of miR-1227-3p promoted cell apoptosis (Fig. 2B). Our result showed that higher expression of miR-1227-3p could prevent the development of FGR.

### Target prediction and bioinformatic analysis of miR-1227-3p

**Figure 3:**
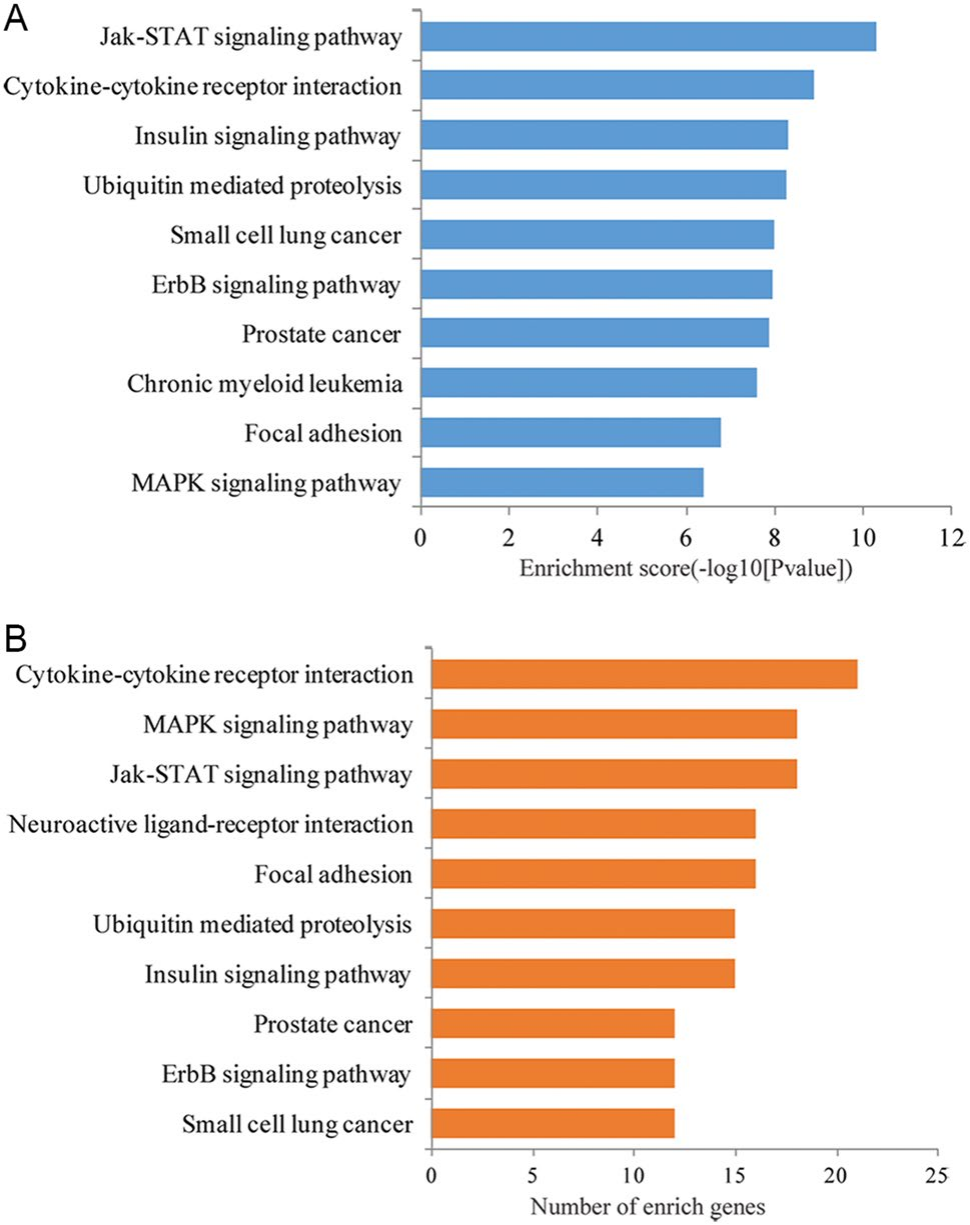
KEGG pathway analysis for the targeted genes of miR-1227-3p. (**A**) KEGG pathway analysis according to the *P* value. (**B**) KEGG pathway analysis according to the number of target genes included.

To examine the function of miR-1227-3p in FGR, we further predicted the target genes of miR-1227-3p using four online predictive systems. As a result, several hundreds of potential targets were identified. Subsequently, we performed the KEGG pathway enrichment analysis for the target genes of miR-1227-3p using DAVID tools. The results indicated that the target genes of miR-1227-3p were significantly enriched in a series of important pathways, such as JAK-STAT signaling pathway, insulin signaling pathway, MAPK signaling pathway, and cytokine–cytokine receptor interaction (Fig. 3). Considering the important role of the insulin signaling pathway in fetal growth and development, we further analyzed the target genes in this pathway. Finally, nine genes (*PRKAB2, AKT1, PIK3R3, MKNK1, SOS1, SOCS3, PRKAA2, PRKX, and PKM2*) were selected as potential target genes for further analysis (Table 3).

**Table 3:** Predicted target genes of miR-1227-3p.

miRNA	Gene	Chromosome	TargetScan	PicTar	DIANA LAB	miRwalk	SUM
miR-1227-3p	PRKAB2	1	√	√	√	√	4
	AKT1	14	√	√	√	√	4
	PIK3R3	1	√	√	√	√	4
	MKNK1	1	√	√	√	√	4
	SOS1	2	√	√	√	√	4
	PRKAA2	1	√	√	√	√	4
	SOCS3	17	√	√	√	√	4
	PRKX	X	√	√	√	√	4
	PKM2	15	√	√	√	√	4

### The target genes of miR-1227-3p were upregulated in FGR-affected placenta tissues

**Figure 4:**
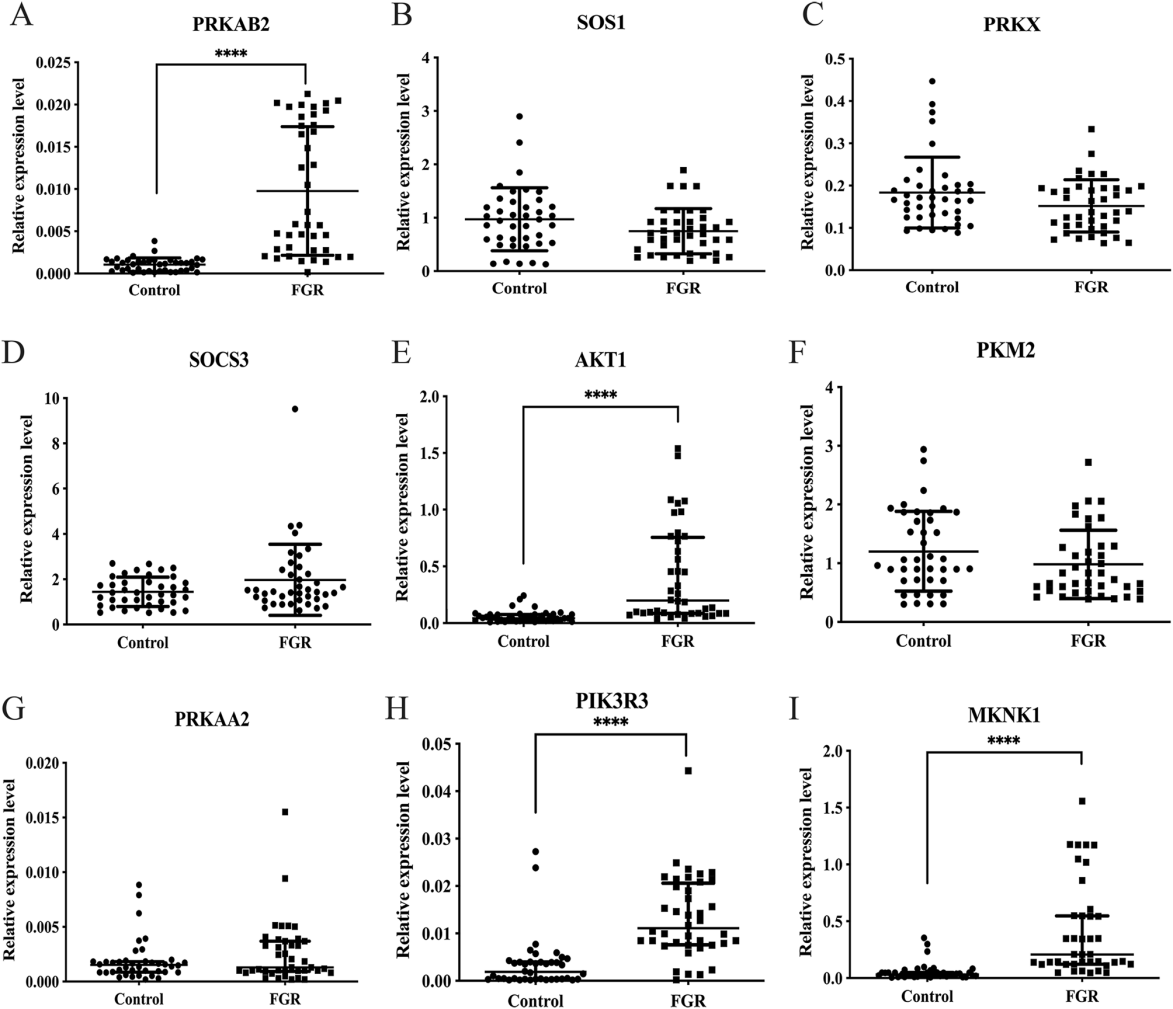
Expression of the predicated target genes of miR-1227-3p in FGR-affected placenta. qRT-PCR was performed to detect the expression of *PRKAB2* (**A**), *PRKX* (**B**) *SOS1* (**C**), *SOCS3* (**D**), *AKT1* (**E**), *PKM2* (**F**), *PRKAA2* (**G**)*, PIK3R3* (**H**), and *MKNK1* (**I**) in 40 FGR-affected placenta and 40 normal placenta. Data are given in Tukey Box plots showing median (−) value. Asterisks denote significant differences from controls (****P < 0.0001).

It is known that miRNA acts as a crucial regulator in gene expression via binding to 3′UTR of target mRNA, and then inhibiting the translation of mRNA or promoting the degradation of mRNA, thus the expression levels of miRNA is negatively correlated with its target genes^18^. Therefore, we further examined the expression level of predicted miR-1227-3p target genes by qRT-PCR using 40 FGR patients and 40 healthy controls. As shown in Fig. 4, the expression levels of *PRKAB2*, *AKT1*, *PIK3R3*, and *MKNK1* were significantly upregulated in FGR-affected placenta tissues compared with normal controls (all *P* < 0.0001). Our results pointed out that miR-1227-3p might be involved in FGR partly through regulating *PRKAB2*, *AKT1*, *PIK3R3*, and *MKNK1*.

## Discussion

A large number of studies have shown that miRNA is involved in preeclampsia, FGR, preterm birth, macrosomia, and other pregnancy-related diseases^19,20^. Cindrova-Davies et al. found miR-21 was significantly upregulated in the placenta of FGR^21^. Recent research demonstrated that miR-206 expression is upregulated in FGR-affected placenta compared with matched normal placenta^22^. Nevertheless, Guo et al. found that the expression of miR-194 was significantly downregulated in the placenta of FGR^23^. However, the key miRNAs involved in FGR and their potential role have not been completely revealed. Therefore, exploring the abnormally expressed miRNAs in FGR-affected placenta, and elucidation of their role and mechanism in FGR is very important. Here, we screened the aberrantly expressed miRNAs in placenta tissues of FGR through miRNA microarray. Our data identified a total of 139 abnormally expressed miRNAs in FGR-affected placenta, and miR-1227-3p was the most highly downregulated miRNA.

The miR-1227-3p gene family mainly includes three members: the precursor hsa-miR-1227, the mature hsa-miR-1227-5p, and the mature hsa-miR-1227-3p. MiR-1227-3p in this study is a 20 nucleotide non-coding single-stranded RNA located on human chromosome 19. There is evidence that miR-1227-3p is overexpressed in many human tumors^24^, and overexpression of miR-1227-3p may be involved in tumor growth by inhibiting apoptosis, promoting cell proliferation, and survival^25,26^. Placental HTR-8/SVneo cells and tumor cells have many similarities, such as rapid proliferation, vascular invasion, rich blood supply and etc.^27^. Increasing evidence demonstrates that the abnormal apoptosis of placental HTR-8/SVneo cells causes the dysfunction of the placenta, and then induces the abnormal development of fetus^28^. However, there is no convincing evidence to introduce the role and mechanism of miR-1227-3p in FGR placental trophoblast dysfunction. Here, our data demonstrated that downregulated miR-1227-3p may promote the cell apoptosis, and inhibited the proliferation of trophoblast cell. Hence, we proposed that miR-1227-3p may be a crucial regulator of FGR development via influencing the function of HTR-8/SVneo cells.

The results of the bioinformatics analysis demonstrated that miR-1227-3p was involved in a series of important pathways, such as insulin signaling pathway, cytokine-cytokine receptor interaction, JAK-STAT signaling, and MAPK signaling. In recent years, the insulin signaling pathway has attracted much attention due to its prominent role in FGR. Peng et al. reported that p53 decreasing significantly relieved the insulin resistance of FGR mice through activating insulin-like growth factor-1 (IGF-1)/AKT signaling^24^. Harris et al. indicated the inhibitory effect of IGF-2 on human placental apoptosis, and knockdown of the IGF2 receptor could induce the decrease of human choriocarcinoma cell proliferation^29^. Given the key role of these pathways in FGR, we speculate that miR-1227-3p may play a crucial role in FGR development through targeting these pathways. Considering the important effect of the insulin signaling pathway in FGR, we demonstrated that the key target genes of miR-1227-3p in the insulin signaling pathway were aberrantly expressed in placenta tissue.

In the present study, our results verified that the expression levels of *PRKAB2*, *AKT1*, *PIK3R3*, and *MKNK1* were significantly increased in FGR-affected placenta. *PRKAB2* is the β regulatory subunits of the AMPK and participates in many cellular biological processes^30,31^. AMPK is a serine/threonine kinase and acts as a major regulator in the protection of the fetal membrane, the homeostasis of maternal and fetal energy, the transportation of nutrients, and the differentiation of the placenta^32^. Studies have found that in the metabolic stress response, AMPK is activated to inhibit the energy expenditure process. However, this protective mechanism can also lead to a decrease in the energy supply of the placental trophoblast and a barrier to the supply of important substances, which promotes the occurrence of FGR^33,34^. *AKT1* is a serine/threonine protein kinase, also known as protein kinase B (PKB). Studies have found that activated *AKT1* can regulate cell apoptosis, cycle operation, invasion, metastasis, and angiogenesis by phosphorylating a variety of substrates^35^. It is indicated that the deregulation of the *AKT1* signaling pathway can lead to placental growth arrest^36^. *PIK3R3* is a regulatory subunit of PI3K. Both *AKT1* and *PIK3R3* are key molecules of the PI3K/AKT cell survival signaling pathway. Abnormal expression of any signal factor in this pathway will affect the action of insulin and form insulin resistance^37^. In the skeletal muscle of low-birth-weight mice, it was observed that the PI3K signal transduction pathway was impaired, and phosphorylation of AKT was reduced, which led to a decrease in the expression of GLUT4 in the cell membrane, thereby affecting the body's uptake and utilization of glucose^38^. Furthermore, *MKNK1* is a downstream effector of MAPK signal transduction, which participated in multiple biological processes, such as cell proliferation, differentiation, transcription regulation, and development. Researches confirmed that the MAPK signaling pathway is widely distributed in placenta tissue. Studies have shown that the activation and expression of p38MAPK may be closely related to the occurrence of FGR by regulating cell proliferation and migration^39^. However, the direct relationships between FGR and these target genes are still unknown. Further studies are needed to illustrate the exact mechanism of these target genes in the development of FGR.

There are some limitations in our study. Firstly, our study only collected a relatively small sample size of placenta tissues. A larger sample size of placenta tissues is required to verify the expression and clinical value of miR-1227-3p in FGR. Secondly, we only verified the most aberrantly expressed miRNA (miR-1227-3p) in placenta tissues after the TLDA screening and analyzed the effect of miR-1227-3p on the proliferation and apoptosis of HTR-8/SVneo cells. Other miRNAs that identified in screening phase may also participant in the development of FGR. The influences of miR-1227-3p on trophoblast cell migration, invasion, and cell cycle are unclear. Lastly, the regulation relationship between miR-1227-3p and its target genes, and whether miR-1227-3p regulates the function of HTR-8/SVneo cells though targeting its target genes are need to be verified. In the future study, we will address these deficiencies, and provide evidence for miR-1227-3p acting as a FGR treatment target.

In conclusion, our data revealed that miR-1227-3p was downregulated in the FGR-affected placenta. Decreased miR-1227-3p may be involved in FGR development by inhibiting trophoblast cell proliferation and promoting apoptosis through regulating genes involved in insulin signaling pathway. MiR-1227-3p may have a potential clinical value in the prevention and treatment of FGR, we need to study further to prove its value in the future.

## Supplementary Information

Supplementary Table S1.

Supplementary Table S2.

## Abbreviations

MAPK: Mitogen-activated protein kinase
PRKAB2: Protein kinase AMP-activated non-catalytic subunit beta 2
AKT1: AKT serine/threonine kinase 1
PIK3R3: Phosphoinositide-3-kinase regulatory subunit 3
MKNK1: MAPK interacting serine/threonine kinase 1
SOS1: SOS Ras/Rac guanine nucleotide exchange factor 1
PRKAA2: Protein kinase AMP-activated catalytic subunit alpha 2
SOCS3: Suppressor of cytokine signaling
PRKX: Protein kinase X-linked
PKM2: Pyruvate kinase M 2
GLUT4: Glucose transporter 4
