# Supplementary material for: miR-1227-3p participates in the development of fetal growth restriction via regulating trophoblast cell proliferation and apoptosis
Source: Sci Rep. 2022 Apr 16;12:6374. doi: 10.1038/s41598-022-10127-w (PMC9013361; doi:10.1038/s41598-022-10127-w)
Supplement: Supplementary file 1 — Supplementary Table S1. [file 41598_2022_10127_MOESM1_ESM.doc]

**Table S1.** List of miRNA in TLDA chips.

| TLDA | miRNA | C_T case_ | C_T control_ | TLDA | miRNA | C_T case_ | C_T control_ |
| --- | --- | --- | --- | --- | --- | --- | --- |
| A | hsa-let-7a-000377 | 26.817 | 28.397 | A | hsa-miR-145-002278 | 26.22 | 27.119 |
| A | hsa-let-7b-002619 | 26.786 | 27.553 | A | hsa-miR-146a-000468 | 22.359 | 22.99 |
| A | hsa-let-7c-000379 | 31.315 | 32.043 | A | hsa-miR-146b-001097 | 25.661 | 25.103 |
| A | hsa-let-7d-002283 | 28.259 | 30.633 | A | hsa-miR-146b-3p-002361 | 40 | 40 |
| A | hsa-let-7e-002406 | 24.275 | 25.408 | A | hsa-miR-147-000469 | 38.312 | 40 |
| A | hsa-let-7f-000382 | 33.099 | 35.828 | A | hsa-miR-147b-002262 | 40 | 40 |
| A | hsa-let-7g-002282 | 25.699 | 26.84 | A | hsa-miR-148a-000470 | 27.357 | 27.184 |
| A | hsa-miR-100-000437 | 24.624 | 24.228 | A | hsa-miR-148b-000471 | 30.85 | 31.288 |
| A | hsa-miR-1-002222 | 29.721 | 32.141 | A | hsa-miR-149-002255 | 25.862 | 26.782 |
| A | hsa-miR-101-002253 | 29.194 | 29.017 | A | hsa-miR-150-000473 | 26.427 | 25.86 |
| A | hsa-miR-103-000439 | 40 | 29.288 | A | hsa-miR-152-000475 | 24.026 | 23.663 |
| A | hsa-miR-105-002167 | 40 | 32.271 | A | hsa-miR-154-000477 | 31.781 | 35.203 |
| A | hsa-miR-106a-002169 | 22.055 | 22.248 | A | hsa-miR-155-002623 | 27.901 | 27.054 |
| A | hsa-miR-106b-000442 | 26.433 | 24.929 | A | hsa-miR-15a-000389 | 25.388 | 29.407 |
| A | hsa-miR-107-000443 | 40 | 40 | A | hsa-miR-15b-000390 | 31.53 | 28.184 |
| A | hsa-miR-10a-000387 | 31.682 | 30.279 | A | hsa-miR-16-000391 | 22.623 | 22.316 |
| A | hsa-miR-10b-002218 | 33.097 | 36.746 | A | hsa-miR-17-002308 | 22.545 | 21.831 |
| A | hsa-miR-122-002245 | 34.354 | 36.349 | A | hsa-miR-181a-000480 | 27.433 | 27.29 |
| A | hsa-miR-125a-3p-002199 | 28.885 | 27.838 | A | hsa-miR-181c-000482 | 33.609 | 35.577 |
| A | hsa-miR-125a-5p-002198 | 28.514 | 28.863 | A | hsa-miR-182-002334 | 40 | 40 |
| A | hsa-miR-125b-000449 | 26.031 | 24.982 | A | hsa-miR-183-002269 | 36.759 | 40 |
| A | hsa-miR-126-002228 | 20.217 | 19.197 | A | hsa-miR-184-000485 | 30.938 | 28.301 |
| A | hsa-miR-127-000452 | 27.158 | 26.82 | A | hsa-miR-185-002271 | 30.3 | 27.894 |
| A | hsa-miR-127-5p-002229 | 40 | 35.703 | A | hsa-miR-186-002285 | 25.836 | 25.031 |
| A | hsa-miR-128a-002216 | 33.504 | 33.898 | A | hsa-miR-188-3p-002106 | 40 | 40 |
| A | hsa-miR-129-000590 | 40 | 40 | A | hsa-miR-18a-002422 | 35.05 | 34.033 |
| A | hsa-miR-130a-000454 | 21.492 | 23.041 | A | hsa-miR-18b-002217 | 40 | 36.659 |
| A | hsa-miR-130b-000456 | 24.644 | 25.186 | A | hsa-miR-190-000489 | 40 | 40 |
| A | hsa-miR-132-000457 | 24.174 | 24.919 | A | hsa-miR-191-002299 | 22.568 | 20.692 |
| A | hsa-miR-133a-002246 | 26.451 | 26.006 | A | hsa-miR-192-000491 | 23.858 | 24.689 |
| A | hsa-miR-133b-002247 | 40 | 40 | A | hsa-miR-193a-3p-002250 | 33.861 | 32.771 |
| A | hsa-miR-135a-000460 | 32.935 | 33.337 | A | hsa-miR-193a-5p-002281 | 26.22 | 27.332 |
| A | hsa-miR-135b-002261 | 26.187 | 25.85 | A | hsa-miR-193b-002367 | 22.112 | 21.061 |
| A | hsa-miR-136-000592 | 39.747 | 35.737 | A | hsa-miR-194-000493 | 29.274 | 28.333 |
| A | hsa-miR-138-002284 | 28.915 | 27.679 | A | hsa-miR-195-000494 | 23.937 | 24.689 |
| A | hsa-miR-139-3p-002313 | 40 | 40 | A | hsa-miR-196b-002215 | 29.91 | 30.648 |
| A | hsa-miR-139-5p-002289 | 28.56 | 29.146 | A | hsa-miR-197-000497 | 30.043 | 30.805 |
| A | hsa-miR-140-3p-002234 | 28.794 | 28.504 | A | hsa-miR-198-002273 | 34.406 | 40 |
| A | hsa-miR-141-000463 | 26.441 | 24.385 | A | hsa-miR-199a-000498 | 29.428 | 29.649 |
| A | hsa-miR-142-3p-000464 | 30.615 | 29.718 | A | hsa-miR-199a-3p-002304 | 25.673 | 24.654 |
| A | hsa-miR-142-5p-002248 | 32.257 | 31.94 | A | hsa-miR-199b-000500 | 33.182 | 32.688 |
| A | hsa-miR-143-002249 | 20.703 | 20.818 | A | hsa-miR-19a-000395 | 29.519 | 29.621 |
| A | hsa-miR-19b-000396 | 23.811 | 24.002 | A | hsa-miR-296-3p-002101 | 40 | 40 |
| A | hsa-miR-200a-000502 | 33.631 | 30.666 | A | hsa-miR-298-002190 | 34.336 | 31.8 |
| A | hsa-miR-200b-002251 | 28.424 | 28.191 | A | hsa-miR-299-3p-001015 | 40 | 40 |
| A | hsa-miR-200c-002300 | 23.766 | 23.102 | A | hsa-miR-299-5p-000600 | 40 | 40 |
| A | hsa-miR-202-002363 | 35.648 | 31.825 | A | hsa-miR-29a-002112 | 20.773 | 20.228 |
| A | hsa-miR-203-000507 | 25.789 | 25.537 | A | hsa-miR-29b-000413 | 30.48 | 29.663 |
| A | hsa-miR-204-000508 | 29.29 | 29.21 | A | hsa-miR-29c-000587 | 23.141 | 22.626 |
| A | hsa-miR-205-000509 | 28.014 | 26.088 | A | hsa-miR-301-000528 | 30.934 | 30.93 |
| A | hsa-miR-208-000511 | 40 | 40 | A | hsa-miR-301b-002392 | 33.441 | 38.91 |
| A | hsa-miR-208b-002290 | 35.315 | 33.194 | A | hsa-miR-302a-000529 | 40 | 40 |
| A | hsa-miR-20a-000580 | 23.491 | 23.142 | A | hsa-miR-302b-000531 | 40 | 40 |
| A | hsa-miR-20b-001014 | 26.537 | 26.424 | A | hsa-miR-302c-000533 | 29.554 | 40 |
| A | hsa-miR-210-000512 | 24.898 | 23.345 | A | hsa-miR-30b-000602 | 24.393 | 26.15 |
| A | hsa-miR-21-000397 | 22.916 | 23.637 | A | hsa-miR-30c-000419 | 23.64 | 25.805 |
| A | hsa-miR-211-000514 | 30.446 | 29.559 | A | hsa-miR-31-002279 | 24.978 | 24.661 |
| A | hsa-miR-212-000515 | 28.845 | 25.654 | A | hsa-miR-320-002277 | 17.713 | 17.667 |
| A | hsa-miR-214-002306 | 25.138 | 24.001 | A | hsa-miR-32-002109 | 32.922 | 32.015 |
| A | hsa-miR-215-000518 | 27.77 | 28.116 | A | hsa-miR-323-3p-002227 | 25.545 | 25.877 |
| A | hsa-miR-216a-002220 | 40 | 40 | A | hsa-miR-324-3p-002161 | 24.534 | 24.18 |
| A | hsa-miR-216b-002326 | 40 | 40 | A | hsa-miR-324-5p-000539 | 25.174 | 25.68 |
| A | hsa-miR-217-002337 | 34.777 | 40 | A | hsa-miR-325-000540 | 40 | 40 |
| A | hsa-miR-218-000521 | 24.257 | 23.754 | A | hsa-miR-326-000542 | 40 | 40 |
| A | hsa-miR-219-000522 | 40 | 40 | A | hsa-miR-328-000543 | 27.737 | 28.79 |
| A | hsa-miR-219-1-3p-002095 | 40 | 40 | A | hsa-miR-329-001101 | 34.003 | 33.314 |
| A | hsa-miR-219-2-3p-002390 | 40 | 40 | A | hsa-miR-330-000544 | 33.305 | 33.836 |
| A | hsa-miR-220-000523 | 40 | 33.693 | A | hsa-miR-330-5p-002230 | 40 | 40 |
| A | hsa-miR-22-000398 | 23.651 | 23.376 | A | hsa-miR-331-000545 | 27.348 | 26.991 |
| A | hsa-miR-220b-002206 | 34.592 | 40 | A | hsa-miR-331-5p-002233 | 30.569 | 31.087 |
| A | hsa-miR-220c-002211 | 40 | 40 | A | hsa-miR-335-000546 | 27.838 | 27.994 |
| A | hsa-miR-221-000524 | 25.044 | 25.569 | A | hsa-miR-337-5p-002156 | 24.125 | 23.492 |
| A | hsa-miR-222-002276 | 17.082 | 18.247 | A | hsa-miR-338-3p-002252 | 40 | 40 |
| A | hsa-miR-223-002295 | 22.131 | 22.244 | A | hsa-miR-339-3p-002184 | 25.255 | 24.505 |
| A | hsa-miR-224-002099 | 24.945 | 25.373 | A | hsa-miR-339-5p-002257 | 40 | 40 |
| A | hsa-miR-23a-000399 | 40 | 31.356 | A | hsa-miR-33b-002085 | 40 | 40 |
| A | hsa-miR-23b-000400 | 40 | 40 | A | hsa-miR-340-002258 | 27.241 | 27.021 |
| A | hsa-miR-24-000402 | 17.098 | 16.829 | A | hsa-miR-342-3p-002260 | 28.852 | 28.883 |
| A | hsa-miR-25-000403 | 27.346 | 28.198 | A | hsa-miR-342-5p-002147 | 33.682 | 40 |
| A | hsa-miR-26a-000405 | 24.265 | 25.179 | A | hsa-miR-345-002186 | 26.486 | 26.443 |
| A | hsa-miR-26b-000407 | 22.281 | 23.513 | A | hsa-miR-346-000553 | 31.499 | 27.927 |
| A | hsa-miR-27a-000408 | 25.482 | 25.487 | A | hsa-miR-34a-000426 | 25.555 | 25.916 |
| A | hsa-miR-27b-000409 | 26.43 | 26.86 | A | hsa-miR-34c-000428 | 32.141 | 33.152 |
| A | hsa-miR-28-000411 | 28.594 | 28.424 | A | hsa-miR-361-000554 | 25.468 | 26.699 |
| A | hsa-miR-28-3p-002446 | 24.962 | 25.95 | A | hsa-miR-362-001273 | 31.109 | 30.487 |
| A | hsa-miR-296-000527 | 33.59 | 34.95 | A | hsa-miR-362-3p-002117 | 30.854 | 30.7 |
| A | hsa-miR-363-001271 | 33.341 | 32.749 | A | hsa-miR-485-3p-001277 | 32.495 | 32.519 |
| A | hsa-miR-365-001020 | 27.599 | 29.244 | A | hsa-miR-485-5p-001036 | 40 | 40 |
| A | hsa-miR-367-000555 | 40 | 40 | A | hsa-miR-486-001278 | 26.835 | 26.251 |
| A | hsa-miR-369-3p-000557 | 28.002 | 29.978 | A | hsa-miR-486-3p-002093 | 31.445 | 31.523 |
| A | hsa-miR-369-5p-001021 | 36.148 | 34.197 | A | hsa-miR-487a-001279 | 40 | 34.178 |
| A | hsa-miR-370-002275 | 29.262 | 26.77 | A | hsa-miR-487b-001285 | 28.035 | 28.437 |
| A | hsa-miR-371-3p-002124 | 27.655 | 27.939 | A | hsa-miR-488-002357 | 40 | 33.227 |
| A | hsa-miR-372-000560 | 28.049 | 25.988 | A | hsa-miR-489-002358 | 24.664 | 25.454 |
| A | hsa-miR-373-000561 | 29.453 | 29.406 | A | hsa-miR-490-001037 | 34.583 | 39.363 |
| A | hsa-miR-374-000563 | 27.464 | 27.738 | A | hsa-miR-491-3p-002360 | 40 | 40 |
| A | hsa-miR-375-000564 | 28.882 | 30.389 | A | hsa-miR-492-001039 | 40 | 40 |
| A | hsa-miR-376a-000565 | 23.833 | 23.594 | A | hsa-miR-493-002364 | 29.242 | 28.405 |
| A | hsa-miR-376b-001102 | 36.876 | 33.686 | A | hsa-miR-494-002365 | 26.014 | 26.645 |
| A | hsa-miR-376c-002122 | 23.21 | 20.372 | A | hsa-miR-499-3p-002427 | 40 | 40 |
| A | hsa-miR-377-000566 | 35.472 | 40 | A | hsa-miR-500-002428 | 30.8 | 29.243 |
| A | hsa-miR-380-3p-000569 | 33.431 | 40 | A | hsa-miR-501-001047 | 40 | 40 |
| A | hsa-miR-381-000571 | 32.65 | 30.987 | A | hsa-miR-501-3p-002435 | 40 | 40 |
| A | hsa-miR-382-000572 | 23.076 | 23.688 | A | hsa-miR-502-001109 | 30.405 | 29.424 |
| A | hsa-miR-383-000573 | 40 | 40 | A | hsa-miR-502-3p-002083 | 32.64 | 31.948 |
| A | hsa-miR-384-000574 | 40 | 40 | A | hsa-miR-503-001048 | 40 | 27.477 |
| A | hsa-miR-409-5p-002331 | 40 | 40 | A | hsa-miR-504-002084 | 40 | 40 |
| A | hsa-miR-410-001274 | 21.513 | 22.02 | A | hsa-miR-505-002089 | 39.303 | 36.167 |
| A | hsa-miR-411-001610 | 26.116 | 25.446 | A | hsa-miR-506-001050 | 40 | 40 |
| A | hsa-miR-412-001023 | 32.855 | 32.989 | A | hsa-miR-507-001051 | 40 | 40 |
| A | hsa-miR-422a-002297 | 33.329 | 32.44 | A | hsa-miR-508-001052 | 37.198 | 34.811 |
| A | hsa-miR-423-5p-002340 | 28.044 | 27.248 | A | hsa-miR-508-5p-002092 | 40 | 40 |
| A | hsa-miR-424-000604 | 27.014 | 25.188 | A | hsa-miR-509-3-5p-002155 | 40 | 40 |
| A | hsa-miR-425-5p-001516 | 28.188 | 28.675 | A | hsa-miR-509-5p-002235 | 31.638 | 40 |
| A | hsa-miR-429-001024 | 40 | 40 | A | hsa-miR-510-002241 | 40 | 40 |
| A | hsa-miR-431-001979 | 24.757 | 24.979 | A | hsa-miR-511-001111 | 26.459 | 25.738 |
| A | hsa-miR-433-001028 | 27.334 | 27.378 | A | hsa-miR-512-3p-001823 | 17.626 | 16.056 |
| A | hsa-miR-448-001029 | 40 | 40 | A | hsa-miR-512-5p-001145 | 26.133 | 25.394 |
| A | hsa-miR-449-001030 | 32.786 | 32.983 | A | hsa-miR-513-5p-002090 | 40 | 40 |
| A | hsa-miR-449b-001608 | 31.928 | 30.809 | A | hsa-miR-515-3p-002369 | 24.786 | 23.368 |
| A | hsa-miR-450a-002303 | 31.057 | 34.535 | A | hsa-miR-515-5p-001112 | 40 | 26.136 |
| A | hsa-miR-450b-3p-002208 | 40 | 40 | A | hsa-miR-516a-5p-002416 | 33.318 | 31.065 |
| A | hsa-miR-450b-5p-002207 | 32.195 | 32.66 | A | hsa-miR-516b-001150 | 24.653 | 23.598 |
| A | hsa-miR-452-002329 | 27.26 | 26.062 | A | hsa-miR-517a-002402 | 14.727 | 14.012 |
| A | hsa-miR-453-002318 | 40 | 40 | A | hsa-miR-517b-001152 | 20.332 | 17.487 |
| A | hsa-miR-454-002323 | 27.085 | 26.95 | A | hsa-miR-517c-001153 | 15.425 | 14.515 |
| A | hsa-miR-455-001280 | 27.055 | 25.268 | A | hsa-miR-518a-3p-002397 | 25.892 | 23.81 |
| A | hsa-miR-455-3p-002244 | 33.212 | 30.615 | A | hsa-miR-518a-5p-002396 | 40 | 32.006 |
| A | hsa-miR-483-5p-002338 | 21.656 | 20.03 | A | hsa-miR-518b-001156 | 26.54 | 25.835 |
| A | hsa-miR-484-001821 | 23.046 | 23.992 | A | hsa-miR-518c-002401 | 27.916 | 24.696 |
| A | hsa-miR-518d-001159 | 29.013 | 27.321 | A | hsa-miR-576-3p-002351 | 32.061 | 30.98 |
| A | hsa-miR-518d-5p-002389 | 27.038 | 25.66 | A | hsa-miR-576-5p-002350 | 40 | 40 |
| A | hsa-miR-518e-002395 | 20.835 | 18.717 | A | hsa-miR-579-002398 | 31.222 | 30.481 |
| A | hsa-miR-518f-002388 | 26.216 | 23.318 | A | hsa-miR-582-3p-002399 | 40 | 40 |
| A | hsa-miR-519a-002415 | 16.313 | 14.867 | A | hsa-miR-582-5p-001983 | 40 | 40 |
| A | hsa-miR-519c-001163 | 25.593 | 24.757 | A | hsa-miR-589-002409 | 40 | 40 |
| A | hsa-miR-519d-002403 | 19.315 | 17.398 | A | hsa-miR-590-5p-001984 | 22.239 | 20.722 |
| A | hsa-miR-519e-002370 | 27.231 | 24.595 | A | hsa-miR-597-001551 | 32.547 | 30.414 |
| A | hsa-miR-520a#-001168 | 25.679 | 25.998 | A | hsa-miR-598-001988 | 30.975 | 29.896 |
| A | hsa-miR-520a-001167 | 27.435 | 24.763 | A | hsa-miR-615-5p-002353 | 40 | 40 |
| A | hsa-miR-520b-001116 | 28.225 | 25.132 | A | hsa-miR-616-002414 | 33.497 | 32.27 |
| A | hsa-miR-520d-5p-002393 | 40 | 29.917 | A | hsa-miR-618-001593 | 40 | 31.935 |
| A | hsa-miR-520e-001119 | 34.537 | 31.366 | A | hsa-miR-624-002430 | 40 | 40 |
| A | hsa-miR-520f-001120 | 29.047 | 26.96 | A | hsa-miR-625-002431 | 29.595 | 27.642 |
| A | hsa-miR-520g-001121 | 24.02 | 23.184 | A | hsa-miR-627-001560 | 33.719 | 31.69 |
| A | hsa-miR-521-001122 | 24.692 | 22.775 | A | hsa-miR-628-5p-002433 | 33.319 | 32.986 |
| A | hsa-miR-522-002413 | 20.088 | 20.422 | A | hsa-miR-629-002436 | 29.968 | 29.714 |
| A | hsa-miR-523-002386 | 28.7 | 24.802 | A | hsa-miR-636-002088 | 29.506 | 28.788 |
| A | hsa-miR-524-5p-001982 | 40 | 40 | A | hsa-miR-642-001592 | 30.257 | 30.683 |
| A | hsa-miR-525-001174 | 24.228 | 24.402 | A | hsa-miR-651-001604 | 40 | 40 |
| A | hsa-miR-525-3p-002385 | 25.388 | 22.965 | A | hsa-miR-652-002352 | 26.546 | 23.941 |
| A | hsa-miR-526b-002382 | 28.398 | 27.016 | A | hsa-miR-653-002292 | 33.102 | 32.609 |
| A | hsa-miR-532-001518 | 24.545 | 23.859 | A | hsa-miR-654-001611 | 28.771 | 26.436 |
| A | hsa-miR-532-3p-002355 | 25.355 | 24.982 | A | hsa-miR-654-3p-002239 | 34.075 | 33.376 |
| A | hsa-miR-539-001286 | 23.758 | 23.507 | A | hsa-miR-655-001612 | 28.438 | 28.893 |
| A | hsa-miR-541-002201 | 40 | 40 | A | hsa-miR-660-001515 | 20.11 | 19.361 |
| A | hsa-miR-542-3p-001284 | 31.142 | 29.956 | A | hsa-miR-671-3p-002322 | 31.096 | 32.65 |
| A | hsa-miR-542-5p-002240 | 40 | 29.93 | A | hsa-miR-672-002327 | 32.376 | 29.203 |
| A | hsa-miR-544-002265 | 38.304 | 34.002 | A | hsa-miR-674-002021 | 40 | 40 |
| A | hsa-miR-545-002267 | 30.888 | 29.183 | A | hsa-miR-708-002341 | 34.412 | 29.421 |
| A | hsa-miR-548a-001538 | 40 | 32.725 | A | hsa-miR-744-002324 | 28.128 | 24.874 |
| A | hsa-miR-548a-5p-002412 | 40 | 40 | A | hsa-miR-758-001990 | 33.788 | 30.623 |
| A | hsa-miR-548b-001541 | 40 | 40 | A | hsa-miR-871-002354 | 40 | 40 |
| A | hsa-miR-548b-5p-002408 | 29.651 | 30.248 | A | hsa-miR-872-002264 | 40 | 40 |
| A | hsa-miR-548c-001590 | 34.77 | 33.488 | A | hsa-miR-873-002356 | 35.14 | 31.81 |
| A | hsa-miR-548c-5p-002429 | 31.571 | 31.805 | A | hsa-miR-874-002268 | 40 | 40 |
| A | hsa-miR-548d-001605 | 40 | 35.06 | A | hsa-miR-875-3p-002204 | 40 | 40 |
| A | hsa-miR-548d-5p-002237 | 32.879 | 32.478 | A | hsa-miR-876-3p-002225 | 40 | 40 |
| A | hsa-miR-551b-001535 | 33.033 | 30.721 | A | hsa-miR-876-5p-002205 | 40 | 40 |
| A | hsa-miR-556-3p-002345 | 40 | 40 | A | hsa-miR-885-3p-002372 | 40 | 40 |
| A | hsa-miR-556-5p-002344 | 40 | 40 | A | hsa-miR-885-5p-002296 | 31.632 | 29.471 |
| A | hsa-miR-561-001528 | 31.272 | 31.094 | A | hsa-miR-886-3p-002194 | 28.663 | 26.305 |
| A | hsa-miR-570-002347 | 40 | 34.433 | A | hsa-miR-886-5p-002193 | 27.627 | 25.019 |
| A | hsa-miR-574-3p-002349 | 26.542 | 24.012 | A | hsa-miR-887-002374 | 40 | 40 |
| A | hsa-miR-888-002212 | 32.2 | 29.512 | A | hsa-miR-9-000583 | 30.096 | 30.956 |
| A | hsa-miR-889-002202 | 26.349 | 25.373 | A | hsa-miR-92a-000431 | 23.758 | 25.352 |
| A | hsa-miR-890-002209 | 33.434 | 32.466 | A | hsa-miR-95-000433 | 31.011 | 33.997 |
| A | hsa-miR-891a-002191 | 34.334 | 33.524 | A | hsa-miR-98-000577 | 29.524 | 40 |
| A | hsa-miR-891b-002210 | 40 | 40 | A | hsa-miR-99a-000435 | 25.39 | 24.814 |
| A | hsa-miR-892a-002195 | 40 | 40 | A | hsa-miR-99b-000436 | 25.93 | 26.285 |
| A | MammU6-001973 | 18.738 | 18.356 |  |  |  |  |
| B | hsa-let-7a#-002307 | 34.158 | 34.349 | B | hsa-miR-1238-002927 | 40 | 40 |
| B | hsa-let-7b#-002404 | 40 | 40 | B | hsa-miR-124#-002197 | 40 | 40 |
| B | hsa-let-7c#-002405 | 40 | 40 | B | hsa-miR-1243-002854 | 32.554 | 34.702 |
| B | hsa-let-7e#-002407 | 38.202 | 40 | B | hsa-miR-1244-002791 | 31.806 | 29.834 |
| B | hsa-let-7f-1#-002417 | 40 | 40 | B | hsa-miR-1245-002823 | 40 | 40 |
| B | hsa-let-7f-2#-002418 | 40 | 36.002 | B | hsa-miR-1247-002893 | 24.511 | 23.936 |
| B | hsa-let-7g#-002118 | 40 | 40 | B | hsa-miR-1248-002870 | 40 | 40 |
| B | hsa-let-7i#-002172 | 40 | 40 | B | hsa-miR-1249-002868 | 40 | 40 |
| B | hsa-miR-100#-002142 | 28.832 | 28.35 | B | hsa-miR-1250-002887 | 40 | 40 |
| B | hsa-miR-101#-002143 | 40 | 40 | B | hsa-miR-1251-002820 | 40 | 40 |
| B | hsa-miR-105#-002168 | 37.243 | 40 | B | hsa-miR-1252-002860 | 40 | 40 |
| B | hsa-miR-106a#-002170 | 40 | 40 | B | hsa-miR-1253-002894 | 40 | 40 |
| B | hsa-miR-106b#-002380 | 29.532 | 29.011 | B | hsa-miR-1254-002818 | 32.145 | 32.519 |
| B | hsa-miR-10a#-002288 | 35.189 | 31.353 | B | hsa-miR-1255A-002805 | 40 | 40 |
| B | hsa-miR-10b#-002315 | 25.75 | 25.678 | B | hsa-miR-1255B-002801 | 28.916 | 28.961 |
| B | hsa-miR-1178-002777 | 40 | 40 | B | hsa-miR-1256-002850 | 35.623 | 34.86 |
| B | hsa-miR-1179-002776 | 34.826 | 33.61 | B | hsa-miR-1257-002910 | 40 | 40 |
| B | hsa-miR-1180-002847 | 30.389 | 28.429 | B | hsa-miR-1259-002796 | 40 | 40 |
| B | hsa-miR-1182-002830 | 40 | 40 | B | hsa-miR-125b-1#-002378 | 29.853 | 29.314 |
| B | hsa-miR-1183-002841 | 29.009 | 27.401 | B | hsa-miR-125b-2#-002158 | 33.733 | 40 |
| B | hsa-miR-1184-002842 | 40 | 34.238 | B | hsa-miR-126#-000451 | 21.327 | 21.545 |
| B | hsa-miR-1197-002810 | 30.802 | 30.719 | B | hsa-miR-1260-002896 | 24.175 | 21.515 |
| B | hsa-miR-1200-002829 | 40 | 40 | B | hsa-miR-1262-002852 | 32.982 | 32.677 |
| B | hsa-miR-1201-002781 | 36.953 | 34.213 | B | hsa-miR-1263-002784 | 40 | 40 |
| B | hsa-miR-1203-002877 | 40 | 40 | B | hsa-miR-1264-002799 | 40 | 40 |
| B | hsa-miR-1204-002872 | 40 | 40 | B | hsa-miR-1265-002790 | 33.71 | 40 |
| B | hsa-miR-1205-002778 | 40 | 40 | B | hsa-miR-1267-002885 | 29.978 | 29.472 |
| B | hsa-miR-1206-002878 | 40 | 40 | B | hsa-miR-1269-002789 | 31.249 | 29.136 |
| B | hsa-miR-1208-002880 | 30.102 | 29.615 | B | hsa-miR-1270-002807 | 33.577 | 32.717 |
| B | hsa-miR-122#-002130 | 40 | 40 | B | hsa-miR-1271-002779 | 35.858 | 32.288 |
| B | hsa-miR-1224-3P-002752 | 40 | 40 | B | hsa-miR-1272-002845 | 40 | 40 |
| B | hsa-miR-1225-3P-002766 | 24.796 | 25.534 | B | hsa-miR-1274A-002883 | 18.666 | 17.735 |
| B | hsa-miR-1226#-002758 | 40 | 40 | B | hsa-miR-1274B-002884 | 15.077 | 14.864 |
| B | hsa-miR-1227-002769 | 25.931 | 29.131 | B | hsa-miR-1275-002840 | 24.05 | 26.66 |
| B | hsa-miR-1228#-002763 | 40 | 40 | B | hsa-miR-1276-002843 | 31.806 | 31.663 |
| B | hsa-miR-1233-002768 | 22.906 | 22.556 | B | hsa-miR-1278-002851 | 40 | 40 |
| B | hsa-miR-1236-002761 | 40 | 40 | B | hsa-miR-1282-002803 | 35.891 | 33.531 |
| B | hsa-miR-1283-002890 | 25.023 | 25.385 | B | hsa-miR-17#-002421 | 40 | 40 |
| B | hsa-miR-1284-002903 | 40 | 40 | B | hsa-miR-181a-2#-002317 | 27.848 | 26.947 |
| B | hsa-miR-1285-002822 | 29.826 | 40 | B | hsa-miR-181c#-002333 | 30.408 | 28.835 |
| B | hsa-miR-1286-002773 | 40 | 40 | B | hsa-miR-182#-000483 | 40 | 40 |
| B | hsa-miR-1288-002832 | 40 | 40 | B | hsa-miR-1825-002907 | 30.09 | 30.339 |
| B | hsa-miR-1289-002871 | 33.669 | 34.225 | B | hsa-miR-1826-002873 | 40 | 40 |
| B | hsa-miR-129#-002298 | 40 | 40 | B | hsa-miR-183#-002270 | 31.423 | 36.12 |
| B | hsa-miR-1290-002863 | 19.039 | 19.034 | B | hsa-miR-185#-002104 | 40 | 40 |
| B | hsa-miR-1291-002838 | 26.274 | 24.91 | B | hsa-miR-186#-002105 | 40 | 40 |
| B | hsa-miR-1292-002824 | 40 | 40 | B | hsa-miR-18a#-002423 | 40 | 34.136 |
| B | hsa-miR-1293-002905 | 40 | 40 | B | hsa-miR-18b#-002310 | 40 | 40 |
| B | hsa-miR-1294-002785 | 40 | 40 | B | hsa-miR-190b-002263 | 31.473 | 31.018 |
| B | hsa-miR-1296-002908 | 40 | 40 | B | hsa-miR-191#-002678 | 30.339 | 29.874 |
| B | hsa-miR-1298-002861 | 40 | 40 | B | hsa-miR-192#-002272 | 40 | 36.068 |
| B | hsa-miR-1300-002902 | 27.765 | 27.433 | B | hsa-miR-193b#-002366 | 27.792 | 26.761 |
| B | hsa-miR-1301-002827 | 40 | 40 | B | hsa-miR-194#-002379 | 40 | 40 |
| B | hsa-miR-1302-002901 | 40 | 40 | B | hsa-miR-195#-002107 | 40 | 40 |
| B | hsa-miR-1303-002792 | 27.94 | 27.65 | B | hsa-miR-196a#-002336 | 40 | 40 |
| B | hsa-miR-1304-002874 | 40 | 40 | B | hsa-miR-19a#-002424 | 40 | 40 |
| B | hsa-miR-1305-002867 | 40 | 40 | B | hsa-miR-19b-1#-002425 | 33.504 | 32.857 |
| B | hsa-miR-130a#-002131 | 40 | 40 | B | hsa-miR-200a#-001011 | 40 | 40 |
| B | hsa-miR-130b#-002114 | 40 | 40 | B | hsa-miR-200b#-002274 | 40 | 40 |
| B | hsa-miR-132#-002132 | 40 | 40 | B | hsa-miR-200c#-002286 | 35.814 | 40 |
| B | hsa-miR-1324-002815 | 40 | 40 | B | hsa-miR-202#-002362 | 40 | 40 |
| B | hsa-miR-135b#-002159 | 30.187 | 30.434 | B | hsa-miR-206-000510 | 31.535 | 29.96 |
| B | hsa-miR-136#-002100 | 21.917 | 21.354 | B | hsa-miR-20a#-002437 | 33.069 | 31.939 |
| B | hsa-miR-138-2#-002144 | 40 | 40 | B | hsa-miR-20b#-002311 | 36.735 | 40 |
| B | hsa-miR-141#-002145 | 40 | 32.359 | B | hsa-miR-21#-002438 | 29.993 | 29.705 |
| B | hsa-miR-143#-002146 | 40 | 40 | B | hsa-miR-213-000516 | 32.891 | 31.809 |
| B | hsa-miR-144#-002148 | 29.904 | 28.42 | B | hsa-miR-214#-002293 | 26.788 | 26.867 |
| B | hsa-miR-144-002676 | 40 | 29.769 | B | hsa-miR-218-1#-002094 | 40 | 40 |
| B | hsa-miR-145#-002149 | 25.246 | 26.096 | B | hsa-miR-218-2#-002294 | 33.99 | 40 |
| B | hsa-miR-146a#-002163 | 40 | 40 | B | hsa-miR-22#-002301 | 27.741 | 26.956 |
| B | hsa-miR-148a#-002134 | 40 | 40 | B | hsa-miR-221#-002096 | 40 | 32.225 |
| B | hsa-miR-148b#-002160 | 31.998 | 31.533 | B | hsa-miR-222#-002097 | 29.367 | 31.798 |
| B | hsa-miR-149#-002164 | 40 | 40 | B | hsa-miR-223#-002098 | 32.472 | 30.886 |
| B | hsa-miR-151-3p-002254 | 28.173 | 25.425 | B | hsa-miR-23a#-002439 | 30.617 | 31.387 |
| B | hsa-miR-151-5P-002642 | 27.128 | 27.609 | B | hsa-miR-23b#-002126 | 40 | 40 |
| B | hsa-miR-154#-000478 | 34.263 | 35.553 | B | hsa-miR-24-1#-002440 | 40 | 40 |
| B | hsa-miR-155#-002287 | 40 | 40 | B | hsa-miR-24-2#-002441 | 40 | 40 |
| B | hsa-miR-15a#-002419 | 40 | 31.118 | B | hsa-miR-25#-002442 | 40 | 40 |
| B | hsa-miR-15b#-002173 | 34.372 | 40 | B | hsa-miR-26a-1#-002443 | 30.455 | 31.911 |
| B | hsa-miR-16-1#-002420 | 40 | 31.171 | B | hsa-miR-26a-2#-002115 | 30.68 | 33.161 |
| B | hsa-miR-16-2#-002171 | 40 | 40 | B | hsa-miR-26b#-002444 | 30.425 | 30.121 |
| B | hsa-miR-27a#-002445 | 27.017 | 27.215 | B | hsa-miR-431#-002312 | 40 | 40 |
| B | hsa-miR-27b#-002174 | 29.665 | 28 | B | hsa-miR-432#-001027 | 34.309 | 35.023 |
| B | hsa-miR-29a#-002447 | 34.556 | 32.979 | B | hsa-miR-432-001026 | 27.517 | 27.219 |
| B | hsa-miR-29b-1#-002165 | 40 | 39.303 | B | hsa-miR-452#-002330 | 35.62 | 40 |
| B | hsa-miR-29b-2#-002166 | 34.199 | 33.219 | B | hsa-miR-454#-001996 | 35.132 | 36.499 |
| B | hsa-miR-302a#-002381 | 31.379 | 40 | B | hsa-miR-483-3p-002339 | 24.351 | 25.424 |
| B | hsa-miR-302b#-002119 | 40 | 40 | B | hsa-miR-488-001106 | 33.323 | 31.528 |
| B | hsa-miR-302c#-000534 | 40 | 40 | B | hsa-miR-497#-002368 | 40 | 40 |
| B | hsa-miR-302d#-002120 | 40 | 40 | B | hsa-miR-497-001043 | 29.154 | 28.826 |
| B | hsa-miR-302d-000535 | 35.121 | 32.297 | B | hsa-miR-500-001046 | 40 | 40 |
| B | hsa-miR-30a-3p-000416 | 25.605 | 24.325 | B | hsa-miR-505#-002087 | 40 | 40 |
| B | hsa-miR-30a-5p-000417 | 21.935 | 20.339 | B | hsa-miR-513B-002757 | 40 | 40 |
| B | hsa-miR-30b#-002129 | 40 | 40 | B | hsa-miR-513C-002756 | 40 | 40 |
| B | hsa-miR-30c-1#-002108 | 40 | 40 | B | hsa-miR-516-3p-001149 | 28.518 | 28.23 |
| B | hsa-miR-30c-2#-002110 | 40 | 40 | B | hsa-miR-517#-001113 | 27.553 | 27.227 |
| B | hsa-miR-30d#-002305 | 32.334 | 28.543 | B | hsa-miR-518c#-001158 | 29.215 | 40 |
| B | hsa-miR-30d-000420 | 23.758 | 21.988 | B | hsa-miR-518e#-002371 | 40 | 40 |
| B | hsa-miR-30e-3p-000422 | 25.49 | 24.175 | B | hsa-miR-518f#-002387 | 40 | 40 |
| B | hsa-miR-31#-002113 | 31.864 | 30.828 | B | hsa-miR-519b-3p-002384 | 25.682 | 23.568 |
| B | hsa-miR-32#-002111 | 40 | 40 | B | hsa-miR-519e#-001166 | 27.837 | 26.635 |
| B | hsa-miR-320B-002844 | 22.998 | 22.85 | B | hsa-miR-520c-3p-002400 | 24.715 | 23.26 |
| B | hsa-miR-335#-002185 | 27.302 | 28.002 | B | hsa-miR-520D-3P-002743 | 24.43 | 22.743 |
| B | hsa-miR-337-3p-002157 | 32.552 | 33.488 | B | hsa-miR-520h-001170 | 28.643 | 27.928 |
| B | hsa-miR-338-5P-002658 | 31.81 | 30.208 | B | hsa-miR-524-001173 | 28.571 | 25.686 |
| B | hsa-miR-33a#-002136 | 35.066 | 32.643 | B | hsa-miR-541#-002200 | 30.413 | 32.412 |
| B | hsa-miR-33a-002135 | 32.666 | 40 | B | hsa-miR-543-002376 | 29.644 | 29.153 |
| B | hsa-miR-340#-002259 | 35.401 | 32.585 | B | hsa-miR-545#-002266 | 40 | 40 |
| B | hsa-miR-34a#-002316 | 40 | 26.162 | B | hsa-miR-548E-002881 | 40 | 40 |
| B | hsa-miR-34b-000427 | 39.298 | 31.457 | B | hsa-miR-548G-002879 | 40 | 40 |
| B | hsa-miR-34b-002102 | 26.781 | 27.456 | B | hsa-miR-548H-002816 | 40 | 40 |
| B | hsa-miR-361-3p-002116 | 40 | 40 | B | hsa-miR-548I-002909 | 33.129 | 39.758 |
| B | hsa-miR-363#-001283 | 40 | 40 | B | hsa-miR-548J-002783 | 40 | 40 |
| B | hsa-miR-367#-002121 | 40 | 40 | B | hsa-miR-548K-002819 | 40 | 40 |
| B | hsa-miR-374a#-002125 | 40 | 35.755 | B | hsa-miR-548L-002904 | 40 | 40 |
| B | hsa-miR-374b#-002391 | 40 | 40 | B | hsa-miR-548M-002775 | 40 | 40 |
| B | hsa-miR-376a#-002127 | 32.037 | 33.909 | B | hsa-miR-548N-002888 | 40 | 40 |
| B | hsa-miR-377#-002128 | 40 | 40 | B | hsa-miR-548P-002798 | 40 | 40 |
| B | hsa-miR-378-000567 | 31.119 | 30.562 | B | hsa-miR-549-001511 | 37.35 | 37.16 |
| B | hsa-miR-378-002243 | 40 | 26.951 | B | hsa-miR-550-001544 | 35.131 | 34.543 |
| B | hsa-miR-380-5p-000570 | 29.179 | 29.008 | B | hsa-miR-550-002410 | 34.429 | 34.075 |
| B | hsa-miR-409-3p-002332 | 24.53 | 23.287 | B | hsa-miR-551a-001519 | 40 | 40 |
| B | hsa-miR-411#-002238 | 35.898 | 32.516 | B | hsa-miR-551b#-002346 | 33.991 | 31.916 |
| B | hsa-miR-424#-002309 | 25.836 | 25.969 | B | hsa-miR-552-001520 | 40 | 40 |
| B | hsa-miR-425#-002302 | 30.213 | 30.057 | B | hsa-miR-553-001521 | 40 | 40 |
| B | hsa-miR-554-001522 | 40 | 40 | B | hsa-miR-614-001587 | 33.16 | 32.081 |
| B | hsa-miR-555-001523 | 40 | 40 | B | hsa-miR-616-001589 | 33.564 | 40 |
| B | hsa-miR-557-001525 | 40 | 40 | B | hsa-miR-617-001591 | 40 | 40 |
| B | hsa-miR-558-001526 | 40 | 40 | B | hsa-miR-620-002672 | 40 | 37.275 |
| B | hsa-miR-559-001527 | 40 | 40 | B | hsa-miR-621-001598 | 40 | 40 |
| B | hsa-miR-562-001529 | 40 | 40 | B | hsa-miR-622-001553 | 28.05 | 27.138 |
| B | hsa-miR-563-001530 | 40 | 40 | B | hsa-miR-623-001555 | 37.397 | 40 |
| B | hsa-miR-564-001531 | 30.448 | 30.082 | B | hsa-miR-624-001557 | 34.052 | 32.836 |
| B | hsa-miR-566-001533 | 40 | 40 | B | hsa-miR-625#-002432 | 26.261 | 26.492 |
| B | hsa-miR-567-001534 | 40 | 36.541 | B | hsa-miR-626-001559 | 40 | 40 |
| B | hsa-miR-569-001536 | 40 | 40 | B | hsa-miR-628-3p-002434 | 30.451 | 31.573 |
| B | hsa-miR-571-001613 | 28.685 | 40 | B | hsa-miR-629-001562 | 30.435 | 29.035 |
| B | hsa-miR-572-001614 | 31.778 | 29.49 | B | hsa-miR-630-001563 | 40 | 34.344 |
| B | hsa-miR-573-001615 | 33.118 | 33.723 | B | hsa-miR-631-001564 | 40 | 40 |
| B | hsa-miR-575-001617 | 40 | 40 | B | hsa-miR-633-001574 | 40 | 40 |
| B | hsa-miR-577-002675 | 40 | 40 | B | hsa-miR-634-001576 | 40 | 40 |
| B | hsa-miR-578-001619 | 40 | 40 | B | hsa-miR-635-001578 | 40 | 40 |
| B | hsa-miR-580-001621 | 40 | 40 | B | hsa-miR-637-001581 | 40 | 40 |
| B | hsa-miR-581-001622 | 40 | 40 | B | hsa-miR-638-001582 | 30.165 | 27.691 |
| B | hsa-miR-583-001623 | 40 | 40 | B | hsa-miR-639-001583 | 32.541 | 30.685 |
| B | hsa-miR-584-001624 | 27.215 | 29.073 | B | hsa-miR-640-001584 | 40 | 35.901 |
| B | hsa-miR-585-001625 | 40 | 40 | B | hsa-miR-641-001585 | 40 | 40 |
| B | hsa-miR-586-001539 | 40 | 40 | B | hsa-miR-643-001594 | 40 | 34.813 |
| B | hsa-miR-587-001540 | 40 | 40 | B | hsa-miR-644-001596 | 40 | 40 |
| B | hsa-miR-588-001542 | 40 | 40 | B | hsa-miR-645-001597 | 33.755 | 32.325 |
| B | hsa-miR-589-001543 | 40 | 39.922 | B | hsa-miR-646-001599 | 40 | 40 |
| B | hsa-miR-590-3P-002677 | 26.714 | 26.153 | B | hsa-miR-647-001600 | 40 | 40 |
| B | hsa-miR-591-001545 | 31.991 | 33.321 | B | hsa-miR-648-001601 | 32.354 | 32.838 |
| B | hsa-miR-592-001546 | 33.468 | 32.706 | B | hsa-miR-649-001602 | 35.511 | 35.184 |
| B | hsa-miR-593-001547 | 40 | 40 | B | hsa-miR-650-001603 | 30.734 | 29.416 |
| B | hsa-miR-593-002411 | 40 | 40 | B | hsa-miR-656-001510 | 26.961 | 28.296 |
| B | hsa-miR-595-001987 | 40 | 40 | B | hsa-miR-657-001512 | 40 | 40 |
| B | hsa-miR-596-001550 | 40 | 29.984 | B | hsa-miR-658-001513 | 40 | 40 |
| B | hsa-miR-599-001554 | 40 | 40 | B | hsa-miR-659-001514 | 29.563 | 28.706 |
| B | hsa-miR-600-001556 | 40 | 40 | B | hsa-miR-661-001606 | 26.932 | 26.055 |
| B | hsa-miR-601-001558 | 32.152 | 28.174 | B | hsa-miR-662-001607 | 40 | 40 |
| B | hsa-miR-603-001566 | 34.722 | 35.744 | B | hsa-miR-663B-002857 | 40 | 40 |
| B | hsa-miR-604-001567 | 32.94 | 33.244 | B | hsa-miR-664-002897 | 28.618 | 27.389 |
| B | hsa-miR-605-001568 | 28.309 | 28.837 | B | hsa-miR-665-002681 | 40 | 40 |
| B | hsa-miR-606-001569 | 40 | 40 | B | hsa-miR-668-001992 | 40 | 40 |
| B | hsa-miR-607-001570 | 40 | 40 | B | hsa-miR-675-002005 | 40 | 40 |
| B | hsa-miR-608-001571 | 40 | 40 | B | hsa-miR-708#-002342 | 40 | 40 |
| B | hsa-miR-609-001573 | 40 | 40 | B | hsa-miR-7-2#-002314 | 40 | 40 |
| B | hsa-miR-613-001586 | 40 | 38.504 | B | hsa-miR-720-002895 | 16.608 | 16.234 |
| B | hsa-miR-744#-002325 | 28.719 | 28.153 | B | hsa-miR-92a-2#-002138 | 40 | 40 |
| B | hsa-miR-765-002643 | 40 | 31.966 | B | hsa-miR-92b#-002343 | 40 | 40 |
| B | hsa-miR-766-001986 | 40 | 40 | B | hsa-miR-93#-002139 | 30.696 | 31.063 |
| B | hsa-miR-767-3p-001995 | 40 | 40 | B | hsa-miR-933-002176 | 40 | 40 |
| B | hsa-miR-767-5p-001993 | 40 | 40 | B | hsa-miR-934-002177 | 32.303 | 32.638 |
| B | hsa-miR-769-3p-002003 | 40 | 40 | B | hsa-miR-935-002178 | 40 | 40 |
| B | hsa-miR-769-5p-001998 | 31.093 | 29.407 | B | hsa-miR-936-002179 | 40 | 40 |
| B | hsa-miR-770-5p-002002 | 32.903 | 40 | B | hsa-miR-937-002180 | 33.025 | 40 |
| B | hsa-miR-802-002004 | 40 | 40 | B | hsa-miR-938-002181 | 40 | 40 |
| B | hsa-miR-875-5p-002203 | 32.103 | 30.033 | B | hsa-miR-939-002182 | 26.844 | 24.746 |
| B | hsa-miR-888#-002213 | 40 | 40 | B | hsa-miR-941-002183 | 40 | 40 |
| B | hsa-miR-892b-002214 | 34.909 | 32.732 | B | hsa-miR-942-002187 | 28.592 | 27.149 |
| B | hsa-miR-9#-002231 | 27.973 | 27.451 | B | hsa-miR-943-002188 | 29.824 | 32.272 |
| B | hsa-miR-920-002150 | 40 | 40 | B | hsa-miR-944-002189 | 29.279 | 28.684 |
| B | hsa-miR-921-002151 | 40 | 40 | B | hsa-miR-96#-002140 | 40 | 40 |
| B | hsa-miR-922-002152 | 40 | 40 | B | hsa-miR-99a#-002141 | 31.871 | 32.394 |
| B | hsa-miR-924-002154 | 40 | 40 | B | hsa-miR-99b#-002196 | 28.952 | 27.99 |
| B | hsa-miR-92a-1#-002137 | 34.401 | 31.798 | B | MammU6-001973 | 18.112 | 15.266 |
