# Supplementary material for: miR-1227-3p participates in the development of fetal growth restriction via regulating trophoblast cell proliferation and apoptosis
Source: Sci Rep. 2022 Apr 16;12:6374. doi: 10.1038/s41598-022-10127-w (PMC9013361; doi:10.1038/s41598-022-10127-w)
Supplement: Supplementary file 2 — Supplementary Table S2. [file 41598_2022_10127_MOESM2_ESM.docx]

Table S2: Forecasting targets of miR-1227-3p

PRKAB2


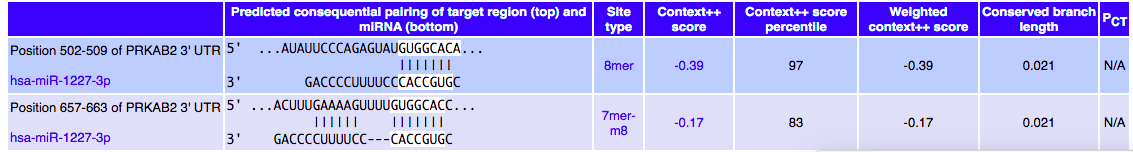


Table S2-1: Schematic representation of the putative miR-1227-3p target site within 3’UTR of the PRKAB2 mRNA in different species.

AKT1
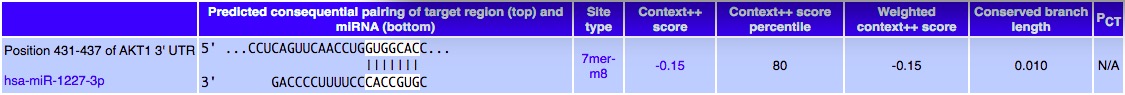


Table S2-2: Schematic representation of the putative miR-1227-3p target site within 3’UTR of the AKT1 mRNA in different species.

PIK3R1
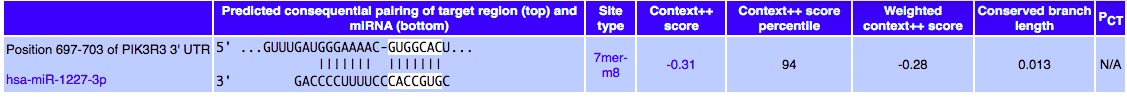


Table S2-3: Schematic representation of the putative miR-1227-3p target site within 3’UTR of the PIK3R1 mRNA in different species.

MKNK1
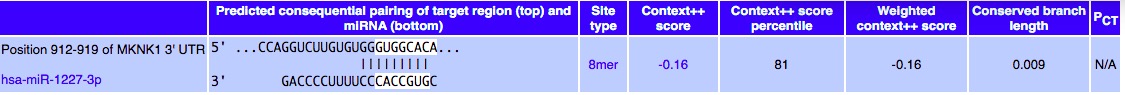


Table S2-3: Schematic representation of the putative miR-1227-3p target site within 3’UTR of the MKNK1 mRNA in different species.
